# Melanogenesis Inhibitory and Antioxidant Effects of *Camellia oleifera* Seed Oil

**DOI:** 10.15171/apb.2017.057

**Published:** 2017-09-25

**Authors:** Puxvadee Chaikul, Tawanun Sripisut, Setinee Chanpirom, Kanchanapa Sathirachawan, Naphatsorn Ditthawuthikul

**Affiliations:** ^1^School of Cosmetic Science, Mae Fah Luang University, Chiang Rai 57100, Thailand.; ^2^Phytocosmetics and Cosmeceuticals Research Group, Mae Fah Luang University, Chiang Rai 57100, Thailand.

**Keywords:** Tea seed oil, Cytotoxicity, Melanogenesis, Antioxidant Cosmetic, cosmetic

## Abstract

***Purpose:*** The study aimed to characterize the fatty acid profile of Camellia oleifera (tea) seed oil and evaluate for cytotoxicity and activities on melanogenesis and antioxidant activity assays in order to utilize as the functional oil.

***Methods:*** The fatty acid profile of oil was analyzed by gas chromatography/mass spectrometry (GC/MS). The cytotoxicity was performed by sulforhodamine B (SRB) assay in B16-F10 melanoma cells and 3T3-L1 cells. The melanogenesis assay, including melanin content and activities of tyrosinase and tyrosinase-related protein-2 (TRP-2), and antioxidant activity were evaluated.

***Results:*** Three major fatty acids of oil were oleic acid (87.93±0.19%), stearic (5.14±0.06%) and palmitic (5.08±0.12%) acids. The non-cytotoxicity of 5% tea seed oil demonstrated the cell viabilities of 94.59±3.41% in B16-F10 melanoma cells and 97.57±1.62% in 3T3-L1 cells. Tea seed oil exhibited the inhibitory activity on melanogenesis assay via inhibition of tyrosinase and TRP-2 activities. The antioxidant activity of 3% tea seed oil appeared the cellular protection with cell viability of 90.38±7.77%.

***Conclusion:*** The results of study have shown the potential utilization of tea seed oil as the functional oil in several products, including health, food and cosmetic products.

## Introduction

*Camellia* spp., the native plants in Eastern Asia, have been cultivated worldwide and compose more than 200 species.^1^ *Camellia oleifera*, a species of tea, is planted for oil-rich seeds. Tea seed oil has reported on several bioactive substances, including fatty acids, polyphenols and sesamin.^2^ Due to the presence of variety of different compounds, tea seed oil has adopted to incorporate in health, food and cosmetic products. In the Chinese traditional medicine, tea seed oil has used in regimens for treatment of stomachache and burning injury.^3^ The oil has employed as edible oil, because of acceptable taste and abundance of antioxidants. For cosmetics, the oil is the rich source of emollient for cosmetic preparation.^4^ However, the study on biological activities of tea seed oil for utilization as the functional agent is limited.

The study aimed to characterize the fatty acid profile of *C. oleifera* seed oil and evaluate for cytotoxicity and activities on melanogenesis and antioxidant activity assays in order to utilize as the functional oil in health, food and cosmetic products.

## Materials and Methods

### Chemicals and reagents 

*C. oleifera* seed oil was purchased from HallStar Company (Illinois, USA). The other reagents were of analytical grade.

### Characterization of fatty acid profile 

The fatty acid profile of oil was analyzed by GC/MS. The esterification of oil was prepared and analyzed as previously described.^5^

### Cytotoxicity assay 

Cytotoxicity assay was performed by SRB assay as previously described.^6^

### Melanogenesis assay 

Melanogenesis assay, including melanin content, tyrosinase and TRP-2 activities, was performed as previously described.^7^

### Antioxidant activity assay 

The antioxidant activity assay was performed as previous method.^7^

### Statistical analysis

Data were expressed as mean ± S.E. One way analysis of variance (ANOVA) and LSD test were used to analyze the results at significant level of *p*-value <0.05.

## Results and Discussion

### Characterization of fatty acid profile

Table 1 is shown the fatty acid profile of tea seed oil. Three major fatty acids were oleic, stearic, and palmitic acids, respectively. Since triacylglycerols consisted of glycerol and three fatty acids are major components of plant oils, the content and types of fatty acids have been responsible for each plant oil characteristics.^8^ The previous studies have been demonstrated the significant correlation of biological activities, including anti-inflammation, wound healing, and moisturizing effect,^9^ and the proportion of unsaturated fatty acids in several functional oils.^10,11^

**Table 1:** Fatty acid profile of tea seed oil

**Fatty acid**	**%**
Myristic acid (C14:0)	0.04±0.00
Palmitic acid (C16:0)	5.08±0.12
Margaric acid (C17:0)	0.10±0.00
Stearic acid (C18:0)	5.14±0.06
Arachidic acid (C20:0)	0.34±0.01
Behenic acid (C22:0)	0.92±0.04
Lignoceric acid (C24:0)	0.25±0.01
Palmitoleic acid (C16:1)	0.19±0.02
Oleic acid (C18:1)	87.93±0.19
Linoleic acid (C18:2)	0.10±0.00

### Cytotoxicity assay

**Figure 1:**
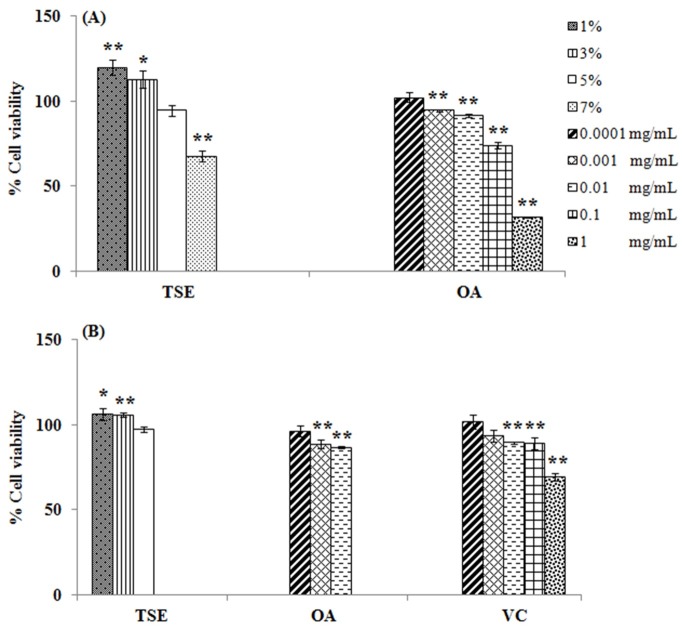
Cytotoxicity assay in B16-F10 melanoma cells (A) and 3T3-L1 cells (B) treated with tea seed oil (TSE), oleic acid (OA) and vitamin C (VC). * indicates significant difference from control (*p<0.05,**p<0.01).

The cytotoxicity assay of tea seed oil was performed in B16-F10 melanoma cells and 3T3-L1 cells. Figure 1 is shown the cytotoxicity assay of tea seed oil and oleic acid. The cytotoxicity of oil and oleic acid depended on treated concentrations. For B16-F10 melanoma cells, 1-5% tea seed oil and 0.0001-0.01 mg/mL oleic acid gave the greater cell viability than 90%, which indicated the non-cytotoxicity. However, the cell viabilities of 7% tea seed oil and 0.1-1 mg/mL oleic acid decreased to less than 80%, which indicated the cytotoxicity. The non-cytotoxic concentrations of oil and oleic acid were evaluated in 3T3-L1 cells in compared to vitamin C, a positive control in antioxidant activity assay (Figure 1). The cell viabilities of 3T3-L1 cells at non-cytotoxic concentrations of oil and oleic acid, and 0.0001-0.1 mg/mL vitamin C were greater than 85%, whereas 1 mg/mL vitamin C decreased cell viability to 69.38±1.99%.

The cytotoxicity of tea seed oil and oleic acid at high concentration may be due to the cell membrane damage. In fact, tea seed oil and oleic acid are lipophilic substances, they may pass through cell membrane and disturb the structure of membrane components.^12^ The cytotoxicity of vitamin C may involve the acidic condition and lead to the inappropriate environment for cell proliferation.^12^

### Melanogenesis assay 

**Figure 2:**
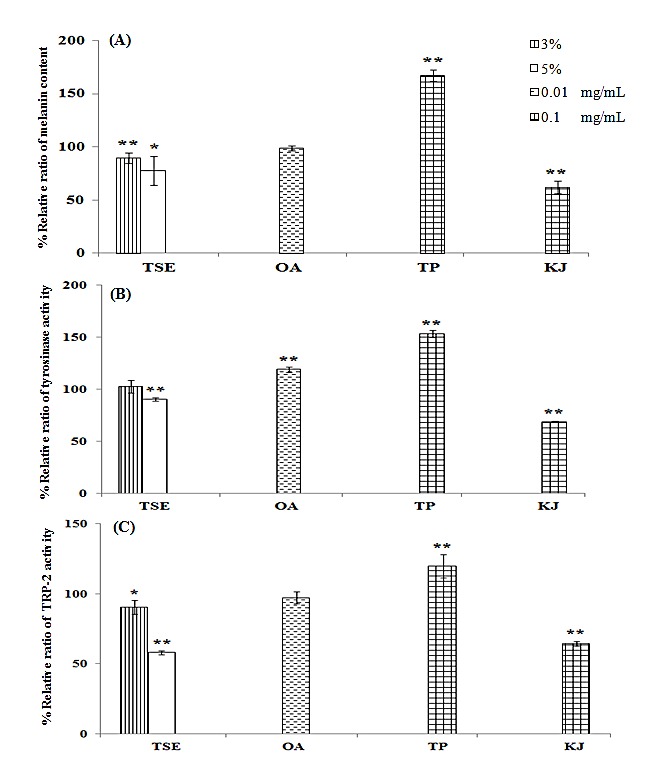
Percentage of relative ratio of melanin content (A), tyrosinase activity (B) and TRP-2 activity (C) in B16-F10 melanoma cells treated with tea seed oil (TSE), oleic acid (OA), theophylline (TP) and kojic acid (KJ). * indicates significant difference from control (*p<0.05, **p<0.01).

Tea seed oil and oleic acid were performed the melanogenesis assay in parallel with theophylline and kojic acid, which were used as positive and negative control, respectively.^7^ The percentage of relative ratios of melanin content, tyrosinase activity, and TRP-2 activity are shown in Figure 2. Tea seed oil significantly decreased melanin content, whereas oleic acid gave the similar effect to control. The melanin contents of theophylline and kojic acid were of 167.09±5.16 and 61.70±5.96%, respectively.

Tyrosinase and TRP-2 activities of tea seed oil were related to melanin content. 5% Tea seed oil significantly decreased the activities of tyrosinase and TRP-2 (*p*-value <0.001). However, tyrosinase activity of oleic acid was not in agreement with effect on melanin content and TRP-2 activity. Tyrosinase activity of oleic acid was significantly increased, whereas TRP-2 activity was similar to control. The tyrosinase and TRP-2 activities of theophylline and kojic acid were correlated with effects on melanin content.

Hyperpigmentation is one of skin problems in Asians that several researchers have investigated the compounds for treatment.^7,13^ Tea seed oil appeared the pigment inhibition via inhibiting of melanogenic enzyme activities. Oleic acid exhibited no effect on melanin content and TRP-2 activity, except tyrosinase. The non-correlation between tyrosinase activity and the pigment regulatory agents have been demonstrated in previous study.^14^ The different melanogenesis effect of oil and oleic acid may be due to the other bioactive compounds of oil, in particularly to polyphenols.^3,15^ The polyphenols of tea seed oil, such as epigallocatechin gallate and catechin gallate,^4^ have shown the inhibitory activity on melanin synthesis and tyrosinase expression.^16^ Theophylline is mediated the effect via cyclic adenosine monophosphate pathway,^17^ whereas kojic acid is mediated via inhibition of tyrosinase in a non-classical manner.^18^

### Antioxidant activity assay 

**Figure 3:**
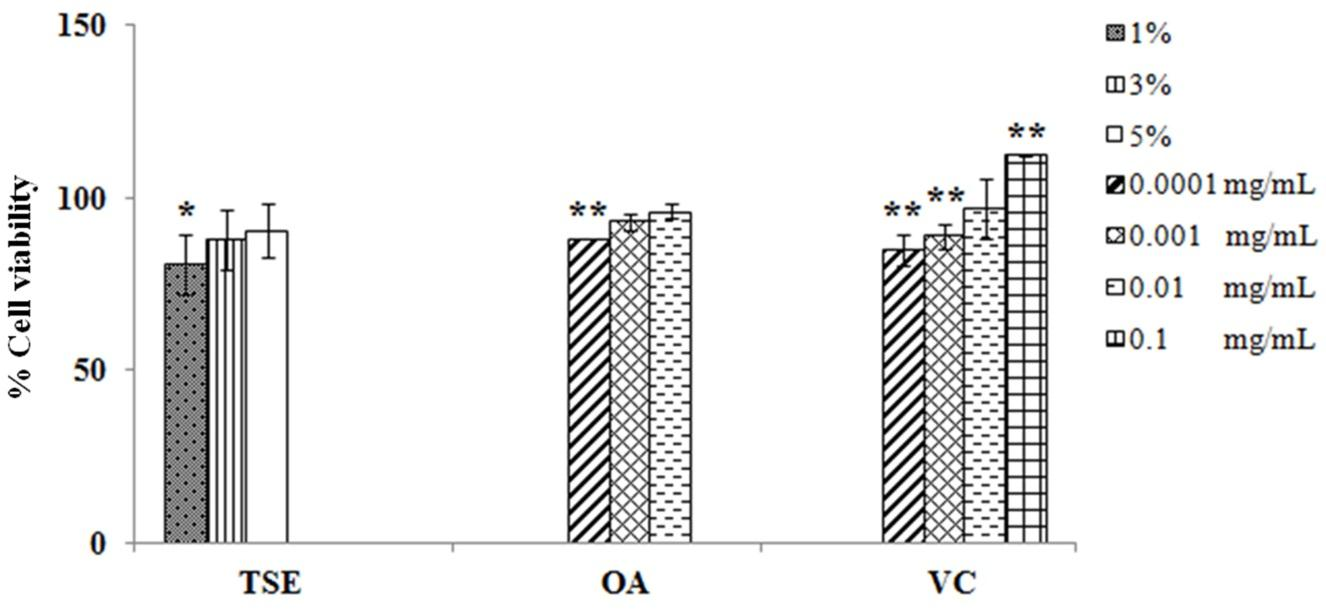
Antioxidant activity assay in 3T3-L1 cells treated with tea seed oil (TSE), oleic acid (OA), and vitamin C (VC). * indicates significant difference from control (*p<0.05,**p<0.01).

Tea seed oil, oleic acid and vitamin C were evaluated for antioxidant activity in 3T3-L1 cells. The antioxidant activity demonstrated the cellular protection after hydrogen peroxide (H_2_O_2_) treatment. The cell viability after treatment with H_2_O_2_ was decreased to 76.50±1.08%. Tea seed oil, oleic acid and vitamin C exhibited the greater cell viability than H_2_O_2_ treatment (Figure 3). 3% Tea seed oil, 0.001 mg/mL oleic acid and 0.01 mg/mL vitamin C were shown the antioxidant characteristics.

The free radicals have correlated with variety of human diseases, the antioxidant can relieve the oxidative stress damage. The antioxidant activity of tea seed oil and vitamin C was in agreement with previous studies.^3,11^ Oleic acid also appeared the antioxidant activity similar to the previous study.^11^ In addition, the other compounds of tea seed oil, including vitamin E, polyphenols, sesamin and compound B, may synergistically play the role in the antioxidant activity.^3^ Vitamin C is mediated activity via reacting with aqueous peroxyl radicals and restoring the antioxidant properties of lipid-soluble vitamin E.^19^

## Conclusion

Tea seed oil has exhibited the oleic acid as a major unsaturated fatty acid, the inhibitory activity on melanogenesis process via inhibition of tyrosinase and TRP-2 activities, and antioxidant activity. The results of study have indicated the potential utilization of tea seed oil as the functional oil in several products, including health, food and cosmetic products.

## Acknowledgments

The work was supported by Mae Fah Luang University [grant number. 58208050020, 2015].

## Ethical Issues

Not applicable.

## Conflict of Interest

The authors declare no conflict of interests.
